# Risks of suboptimal and excessive thyroid hormone replacement across ages

**DOI:** 10.1007/s40618-023-02229-7

**Published:** 2023-11-28

**Authors:** U. Feldt-Rasmussen, G. Effraimidis, S. Bliddal, M. Klose

**Affiliations:** 1 Department of Medical Endocrinology and Metabolism, Rigshospitalet, Copenhagen University Hospital, Copenhagen, Denmark; 2 Institute of Clinical Medicine, Faculty of Health and Clinical Sciences, Copenhagen University, Copenhagen, Denmark; 3 Department of Endocrinology and Metabolic Diseases, Larissa University Hospital, Larissa, Greece; 4 Faculty of Medicine, School of Health Sciences, University of Thessaly, Larissa, Greece; 5 Department of Medical Endocrinology and Metabolism PE 2132, Rigshospitalet, National University Hospital, Blegdamsvej 9, 2100 Copenhagen, Denmark

**Keywords:** Hypothyroidism, Replacement, Non-communicable disease, Suboptimal treatment

## Abstract

**Background:**

Hypothyroidism is prevalent at all ages and represents a non-communicable disease with preventable consequences.

**Method:**

Narrative review.

**Review:**

In children and adolescents, the most devastating consequences of undertreatment with levothyroxine (LT4) are poor growth and development. Delayed treatment in congenital hypothyroidism can lead to permanent brain damage. In young to middle-aged adults, symptoms are often overlooked, and treatment delayed by many years. The resulting consequences are also at this age group compromised brain and physical function but less severe and partly reversible with treatment. The under-treated condition often results in a higher risk of, e.g., increased cardiovascular disease burden, obesity, hypertension, poor physical capacity, and poor quality of life. Excessive replacement is at all adult age groups associated with increased risk of cardiac death, osteoporosis, loss of muscle function, psychological instability and poor quality of life. In young fertile women, the consequences of undertreatment with LT4 are subnormal fertility, recurrent pregnancy loss, compromised fetal growth, and neurocognitive development. On the other hand, excessive LT4 treatment has been related to gestational hypertension, preeclampsia and preterm birth. In the elderly, care must be given to avoid confusing a slightly high age-related serum TSH with requirement for LT4 treatment in a truly hypothyroid patient. Excessive LT4 treatment in patients of high age is associated with an increased mortality.

**Conclusion:**

Suboptimal and excessive LT4 replacement of the preventable non-communicable disease hypothyroidism requires more focus from the healthcare system and from the global political systems to prevent the personally devastating and socioeconomically challenging consequences.

## Introduction

Hypothyroidism (or myxedema) is a common non-communicable endocrine disorder worldwide, depending on the composition of the populations’ age, sex, race, genetics, food intake, environmental factors such as iodine and selenium intake as well as the specific diagnostic criteria and many other influences [1]. In typical cases of overt hypothyroidism, the diagnosis is not difficult, but there are several gray zones such as how to manage the milder or subclinical cases, how to distinguish influence from non-thyroidal illness, and the differences in reference ranges across ages.

Thyroxine (T4) was chemically identified in 1927 [2] and the second thyroid hormone—triiodothyronine (T3) 25 years later [3]. L-thyroxine (LT4) has been commercially available since the 1950ies and remains the cornerstone in treatment of hypothyroidism [4, 5], despite several challenges mainly including that of patients and patient organizations wishing to shift to combination therapy with T3 [1] or to revert to desiccated thyroid preparations [1], none of which is recommended in current guidelines and not used abundantly in, e.g., Denmark [4].

The aim of this narrative review is to give evidence for the long-term efficacy of LT4 and the risks and consequences of both suboptimal and excessive replacement throughout different ages and patient populations.

## Causes of hypothyroidism

Globally, the main cause for spontaneous hypothyroidism is still overt iodine deficiency or partial iodine deficiency for special conditions such as pregnancy. In populations of sufficient or moderately iodine deficiency the pattern of hypothyroidism is different. Thus, a large population study in Denmark reported that the most common subtype (present in 84.4% of patients) was spontaneous (presumably autoimmune) hypothyroidism, followed by post-partum (4.7%) and amiodarone-induced hypothyroidism (4.0%). Less common causes were subacute thyroiditis (1.8%), previous radiation or surgery (1.8%) to the thyroid gland, congenital (1.6%) and lithium-associated (1.6%) thyroid failure. Iatrogenic causes have become more frequent due to various immunotherapies [6]. Rarer causes include central (secondary) hypothyroidism, thyroid hormone resistance, overtreatment with antithyroid drugs, effects of other drugs, ingestion of food containing goitrogens (e.g., vegan food), and therapeutic or environmental irradiation [1].

## Physiology of thyroid function and diagnosis of hypothyroidism

The diagnosis of overt hypothyroidism can sometimes be based on the phenotype, but in most cases biochemical measurement of thyroid function variables is necessary and recommended, particularly in subtle cases. In some situations, it will also be needed to measure supportive biomarkers [7]. Thyroid hormone secretion is regulated in a negative feedback manner by pituitary thyrotropin (TSH), which in turn is stimulated by the hypothalamic TSH releasing hormone (TRH) [1]. Obtaining a normal euthyroid state is, thus, a delicate balance between the hypothalamus–pituitary function and that of the thyroid gland, regulated in a logarithmic (TSH)/linear (T4 and T3) way.

The reliability of the biochemical measurements on the one hand, and the cut-off values of the hormone measurements between normal and hypothyroid persons on the other, are therefore crucial for a correct diagnosis. Due to the above-mentioned log/linear relationship, serum TSH is the most sensitive variable to assess thyroid function, The diagnosis of primary hypothyroidism is nevertheless based on biochemical demonstration of low circulating T4 in the face of an elevated serum TSH beyond the reference limits of normal people. A universally accepted reference range has been suggested as 0.4–4.0 mU/L, but is unfortunately too simplistic, since there are huge variations among populations in terms of environmental influence such as iodine intake, age, genetics, and many others. Serum TSH is, thus, higher in areas of both overt and partial iodine deficiency [8], very high in neonates [9], variable during pregnancy according to gestation [10], increasing at high age [11] and higher in some genetic population groups such as Ashkenazy Jews [12]. Circulating thyroid hormones show similar reference range differences although less well explored. Consequently, current reference ranges could potentially lead to inappropriate commencement of treatment in older individuals and in certain populations with other genetic traits. However, they could also result in inappropriate treatment at all ages. There is no evidence to support the general use of age-appropriate reference intervals, nor to understand the impact of thyroid hormone variations in younger individuals.

On top of these physiological challenges, there are also a number of pitfalls in the biochemical measurements of the thyroid-related hormones [6, 13, 14]. Although most of them are rare, it is important to know of their existence since they may otherwise result in wrong diagnoses and treatment [6, 15, 16]. Finally, a number of concomitant drug intakes can compromise the absorption of levothyroxine [6, 17] as can gastrointestinal diseases such as coeliac disease, pernicious anemia, ulcerative colitis [18], and many more. Other drug intakes can also influence the thyroid-related hormone measurements as well [6].

## Suboptimal and excessive treatment in children and adolescents

Severe untreated congenital hypothyroidism will result in cretinism with blunted growth, very low IQ, generally failed neurological development and psychological disturbances. In the Western part of the world, such cases are irradicated by introduction of a neonatal TSH screening program. However, despite the available dried blood spots technology since the early 1970s, approximately 70% of the world’s population does not have access to it. Therefore, children still develop cretinism [19]. Congenital hypothyroidism usually cannot be diagnosed on clinical grounds until the child is several months old. The consequent treatment delay therefore leaves these children with permanent neurological damage. To increase the early detection of milder cases of congenital hypothyroidism, the screening TSH level has been lowered, and free T4 measurements have in some countries been added to diagnose also congenital central hypothyroidism [20, 21].

The causes of hypothyroidism occurring later in childhood and in adolescents include iodine deficiency, subtle congenital defects in, e.g., thyroid hormone synthesis, hereditary thyroid disorders (thyroid hormone resistance), autoimmune thyroid disease, and potentially environmental endocrine disruptors. Later occurrence is usually associated with milder symptoms, but also results in impaired growth, behavioral problems and symptoms of attention-deficit/hyperactivity disorder (ADHD), failure of obtaining a proper education and, thus, very often failure to become independent adults. This disorder, therefore, also has high socioeconomic consequences for the individual and society, which might be prevented or reversed if treated early with LT4 [22].

Consequences of excessive LT4 replacement are unfortunately not well explored in this age group (Table 1).

**Table 1:** Overview of consequences and risks of suboptimal levothyroxine treatment

Age group	Consequences and risks of undertreatment LT4	Consequences and risks of overtreatment LT4
Intrauterine and neonatal	Poor growth and brain development	Accelerated or poor growth and brain development
Children and adolescents	Poor growth and general development	Poor growth and general development
	Poor development of teeth and bones	Poor development of bones and teeth
	Cognitive disturbance	Cognitive disturbance
	Behavioral disturbance (ADHD)	Behavioral disturbance (ADHD)
Young adults	Poor mental function, ADHD or other behavioral conditions	Psychological instability, ADHD, manic behavior
	Reduced cardiac output	Accelerated heart rhythm, incl atrial fibrillation
	Decreased physical capacity	Decreased physical activity and functioning
	Low cardiac output	Muscle weakness
	Muscle weakness	Hair loss
	Reduced lung function	Increased heat production and sweating
	Hypertension	Increased metabolism of many medications
	High total and LDL cholesterol	Poor quality of life
	Increased cardiovascular disease burden	Unintended weight loss
	Hair loss	Increased cardiovascular mortality risk
	Dry skin	Vitamin deficiencies
	Low production of many substances, e.g., hemoglobin and glucocorticoids	
	Low glomerular filtration rate	
Fertile females	Menstrual disturbances	Menstrual disturbances
	Infertility	Infertility
	Pregnancy loss	Pregnancy loss
	Recurrent pregnancy loss	Recurrent pregnancy loss
	Post-partum thyroiditis	Preeclampsia
		Gestational hypertension
		Post-partum thyroiditis
All age groups	Non-thyroidal illness	Depression, psychological instability, ADHD, manic behavior
	Proteinuria, other protein loss	Accelerated heart rhythm, incl atrial fibrillation
	Decreased general metabolism	Decreased physical activity and functioning
	Anemia	Muscle weakness
	Lowered substance production and degradation	Hair loss
	Slow elimination of medications	Increased heat production and sweating
	Malabsorption	Increased metabolism of many medications
	Increased sympathetic tone	Poor quality of life
	Vegan food	Unintended weight loss
	Poor quality of life and general work- functioning	Increased cardiovascular mortality risk
		Vitamin deficiencies
		Palpitations and constant fidgeting
Elderly, old age	As young adults and all age groups +	As young adults and all age groups +
	Heart failure	Heart failure
	Poor cognition, slow cerebration	Poor cognition,
	Higher mortality rate	Higher mortality rate
	Increased cardiovascular morbidity	Increased cardiovascular morbidity

Footnotes to tables should be indicated by superscript lower-case letters (or asterisks for significance values and other statistical data) and included beneath the table body

## Suboptimal and excessive treatment in adults after puberty until old age

Young or middle-aged adults are also at risk of underdiagnosis and undertreatment for hypothyroidism mostly due to very non-specific symptomatology and few typical features [1]. Since thyroid hormones are responsible for cellular metabolism in all mammalian organs, it is not surprising that symptoms can occur from all organ systems if thyroid hormones are reduced even only slightly. The risks of undertreatment of a diagnosed hypothyroid state are related to poor mental function, ADHD or other behavioral conditions, reduced cardiac output, and decreased physical capacity due to low cardiac output combined with muscle weakness and reduced lung function (1). A reflex overstimulation of catecholamines leads to hypertension, which together with obesity subsequently leads to an increased cardiovascular disease burden. Patients often have hair loss, dry skin, and constipation, and due to a slow metabolism, patients have a low production of many substances, e.g., hemoglobin and adrenal glucocorticoids. Conversely, there is a low degradation of many substances giving rise to, e.g., elevated liver enzymes. Furthermore, patients with under-treated hypothyroidism have a low glomerular filtration rate. Many drugs may have a slow metabolism and thereby risk accumulation or direct poisoning. All of these symptoms and signs result in a poor quality of life and general work-functioning [1].

Other factors may also increase the risk, e.g., protein loss in proteinuria causing urinary loss of LT4 and, therefore, requires an increase of the LT4 replacement dosage, whereas several other drugs or food components can interfere with the gastric absorption of LT4.

## Suboptimal and excessive treatment in women during reproductive age

Women of reproductive age face a number of risks of both over- and undertreatment due to challenges related to interpretation of thyroid function tests and varying needs for LT4 treatment. Women with a higher concentration of estrogen, e.g., by use of oral contraceptives or during pregnancy, will have an increased concentration of thyroid hormone binding proteins, which will cause an increase in the total T4 concentration. This will interfere with most immunoassays measuring free T4 as they are unable to sufficiently correct for the extremes found in the high end of the correction curves. Thus, there is a risk of misinterpreting the free T4 concentrations and thereby under- or overtreating the women and correct management will require additional measures of thyroid function. Specifically in pregnancy, the LT4 treatment is not only important to the woman, but also to secure a sufficient placental transfer of T4, which is needed for fetal development and to reduce adverse pregnancy outcomes such as pregnancy loss and preterm birth [23, 24]. To avoid undertreatment, the LT4 dosage should be increased to mimic the physiological increase in T4 in pregnancy [7, 25].

Furthermore, undertreatment of hypothyroid women will often lead to irregular menstrual periods and infertility, which is also the reason for the internationally recommended screening for thyroid dysfunction of all women referred to fertility treatment [25, 26]. Hypothyroid women treated with LT4 will need an increased dose prior to fertility treatments involving controlled ovarian stimulation as such stimulation induces an acute increased TBG production. Women in fertility treatment with untreated subclinical hypothyroidism may need LT4 substitution prior to fertility treatment to support the critical phase of implantation and early pregnancy [27, 28]. On the other hand, slight aberrations in thyroid function may not need to be treated given the risk of overtreatment, which could also lead to (iatrogenic) preeclampsia and preterm birth [28]. Noteworthy, there is no evidence to support a benefit to offspring IQ by LT4 treatment of subclinical hypothyroidism or hypothyroxinemia [29, 30].

Finally, the post-partum period requires changes in dosage to return to pre-pregnancy dosage. However, in women with remaining thyroid gland function and thyroid autoimmunity, there is a risk of post-partum thyroiditis with an initial destructive thyroiditis with release of thyroid hormone and hyperthyroidism, followed by a hypothyroid phase until regeneration of the thyroid function (if any left). The hypothyroid phase requires an increased dose of LT4, which is often transitory, but may become permanent. Monitoring in the post-partum phase is therefore important to secure optimal treatment, which is even more important as there is a risk of suboptimal treatment resulting in a limited capacity to take care of their child due to fatigue, and risk getting a diagnosis of post-partum depression. Women with unexpected symptoms post-partum should, therefore, also have their thyroid function tested to discover and treat hypothyroidism [31].

## Suboptimal and excessive treatment of the elderly and people with cardiac diseases

TSH reference ranges in the elderly must be evaluated in the light of the age-dependent shift in serum TSH distribution toward higher concentrations with increasing age. Studies in several populations (Americans, Scottish, Ashkenazi Jews, Chinese) have shown progressively increasing TSH concentrations with age. The 97.5th percentiles of the reference population are considerably higher in the elderly (older than 70 years old) than in younger adults [32–35], which is not explained only by higher prevalence of thyroid autoimmunity [36, 37]. This phenomenon might reflect an age-related alteration in the TSH set point and/or reduced TSH bioactivity and/or reduced sensitivity of the thyroid gland to TSH, since longitudinal data suggest an inter-individual age-dependent TSH increment not associated with a decline in free T4 [36]. Although an association between increased serum TSH and/or lower levels of thyroid hormones and extended life span have been observed, it is still unclear if it is a positive adaptation or a progressive, negative deterioration [38].

A recent large cohort study found that approximately 1/3 of older hypothyroid patients on LT4 treatment had taken at least one medication that interferes with thyroid hormones [39]. In addition, common chronic conditions such as heart, kidney, liver disease, diabetes, major depression, as well as low caloric intake, which are more prevalent in the elderly, may result in changes in thyroid function as part of the euthyroid sick syndrome. For these reasons, a detailed careful review of drugs and comorbidities should always be conducted before an older individual with hypothyroidism treated with LT4 is classified as under-treated [36]. The age-dependent shift in serum TSH distribution toward higher concentrations with increasing age and an observed relationship between longevity and lower thyroid function prompted experts to recommend a higher TSH level target of, e.g., 6 mIU/L in a 70-year-old patient treated with LT4 [40] considering other factors (drugs, comorbidities) affecting the thyroid function tests.

However, several studies showed insufficiently treated overt and/or subclinical hypothyroidism as well as excessively replaced hypothyroid patients associated with increased early mortality risk in older patients [41–44]. Furthermore, a recent study found that the cardiovascular mortality risk increased progressively with higher TSH, especially in the older adults [14]. These studies used different criteria and different number of TSH measurements regarding the classification of under-treated patients, but in any case, these findings highlight the importance for further studies on the optimal TSH target in older hypothyroid patients.

The profound negative effect of hypothyroidism, and the beneficial effect of LT4 therapy on cardiac function should be taken into consideration when planning thyroid hormone therapy [36, 45, 46]. A recent retrospective cohort study of more than 150,000 patients with incident hypothyroidism of whom 97% received LT4 therapy during the follow-up period, showed that there was an association between the highest TSH concentration and increased risk of heart failure both in patients younger and older than 65 years; whereas, the increased risk of ischemic heart disease remained significant only in patients ≤ 65 years [43]. The risk of stroke/transient ischemic attack was not increased in any patients. These findings are, thus, not conclusive concerning the level of LT4 treatment in the hypothyroid elderly, particularly when considering the serious risks of excessive LT4 replacement in the elderly (Table 1).

Recently, a comprehensive meta-analysis including 55 studies and a total of 1,898,314 subjects, concluded that the presence of overt hypothyroidism is associated with an increased risk of myocardial ischemia (13%), myocardial infarction (15%), arrhythmias (96%) and overall mortality (25%) when compared to euthyroidism [47]. In another paper, a meta-analysis involved 7 studies and 31,138 subjects concluded that the presence of overt hyperthyroidism increased overall mortality by 13% and the mortality for cardiovascular causes by 21% [48]. Another more recent meta-analysis including 37 studies and enrolling 113,393 hyperthyroid subjects confirmed that overt hyperthyroidism increased the risk of ischemic heart disease, stroke, and cardiovascular mortality [49].

## Suboptimal and excessive LT4 replacement in other special situations

In hypothalamus–pituitary–hypothyroidism or central/secondary hypothyroidism, the thyroid hypofunction is due to a failing pituitary production of biologically active TSH. Unfortunately, the biologically inactive TSH molecule is measurable by the TSH immunoassays and therefore useless in the diagnosis of hypothyroidism as well as monitoring of replacement [50]. Total and free T4 measurements (free T4 estimates) are thus the only useful tools by current methodology [51]. In case of multiple pituitary hormone deficiencies, the situation becomes more complex, e.g., in women on estrogen replacement therapy (or on oral contraceptive drugs), except worse, since TSH cannot be used [52]. These women are, therefore, at high risk of suboptimal replacement with a consequent worse metabolic profile [53].

Patients in intensive care units (ICU) are also particularly problematic because they often cannot take tablets orally. In these situations, patients need other administration routes either i.v LT4 or oral liquid formulation [54]. In the monitoring of the biochemical thyroid function, it is important to realize that all patients in ICU have non-thyroidal illness (NTI) [55] with uninterpretable thyroid function tests. The best is to continue the regular replacement dose independent of the false thyroid function measurements to avoid both suboptimal and excessive replacement [56].

## Conclusions

Hypothyroidism is very prevalent at all age groups and represents a non-communicable disease in which the risks and consequences are preventable. However, the diagnosis is often delayed for long periods with resulting consequences for the individual and the society. Excessive LT4 overdosing also infers serious risks such as osteoporosis, cardiac arrythmias and heart failure, muscle wasting, poor quality of life, and cognitive disturbances.

Hypothyroidism as a non-communicable disease is not part of any governmental focus. Suboptimal management of the preventable hypothyroidism resulting in a risk of both under- and excessive treatment, therefore, requires more focus both from caretakers in the healthcare system, but also from the global political systems to prevent the personally devastating and socioeconomically challenging consequences.
